# Rush Medical College

**Published:** 1852-01

**Authors:** 


### RUSH MEDICAL COLLEGE.

There will be a course of Anatomical Lectures, with Dissections and Demonstrations, in this Institution, by J. W. Freer, M. D., Demonstrator of Anatomy, to commence on the 23d of February next, and continue six weeks. Terms $5.

Clinical instruction will be given in the Hospital.

